# Enhancement of *Epimedium* Fried with Suet Oil Based on *in Vivo* Formation of Self-Assembled Flavonoid Compound Nanomicelles 

**DOI:** 10.3390/molecules171112984

**Published:** 2012-11-01

**Authors:** Li Cui, E Sun, Zhen-Hai Zhang, Xiao-Bin Tan, Ying-Jie Wei, Xin Jin, Xiao-Bin Jia

**Affiliations:** 1 Key Laboratory of New Drug Delivery System of Chinese Meteria Medica, Jiangsu Provincial Academy of Chinese Medicine, 100 Shizi Road, Nanjing 210028, Jiangsu, China; Email: cuili2008516@126.com (L.C.); sune0825@yahoo.com.cn (E.S.); davidpharm@yeah.ne (Z.-H.Z.); njtxb@hotmail.com (X.-B.T.); wyj970@163.com (Y.-J.S.); jinxin871211@163.com (X.J.); 2 Nanjing University of Chinese Medicine, Nanjing 210046, Jiangsu, China

**Keywords:** *Epimedium*, suet oil, self-assembled nanomicelles, icariin, Caco-2 cell monolayers, single-pass rat intestinal perfusion

## Abstract

The purpose of this work was to research the enhancement of *Epimedium* fried with suet oil based on the *in vivo* formation self-assembled flavonoid nanomicelles. Taking icariin as the representative, under the action of suet oil, self-assembled nanomicelles were prepared under simulated gastrointestinal tract conditions and were characterized by dynamic light scattering and transmission electron microscopy (TEM). The experiments with icariin self-assembled nanomicelles without suet oil were done according to the above. The influence of suet oil on the transportation of icariin across Caco-2 cell monolayers and the absorption in rat intestine of self-assembled nanomicelles were evaluated. The particle size of icariin self-assembled nanomicelles with suet oil was smaller than without suet oil. The nanomicelles seemed to be monodisperse spherical particle with smooth surfaces. The icariin entrapment efficiency of self-assembled nanomicelles with suet oil was increased from 43.1% to 89.7%. In Caco-2 cell monolayers, the absorptive permeability, secretory permeability and efflux ratio of icariin self-assembled nanomicelles with suet oil was 1.26 × 10^−6^ cm/s, 5.91 × 10^−6^ cm/s and 4.69, respectively, while that of icariin self-assembled nanomicelles without suet oil was 0.62 × 10^−6 cm/s^, 3.00 × 10^−6^ cm/s, and 4.84, respectively. In rat intestinal perfusion experiments, the permeability coefficient of icariin self-assembled nanomicelles with suet oil in duodenum was higher than the value of icariin self-assembled nanomicelles without suet oil (*p* < 0.05). With the action of suet oil, icariin self-assembled nanomicelles were more stable and the entrapment efficiency was higher than that without suet oil, which could increase the solubility of icariin and improve its intestinal absorption. Therefore, suet oil plays a role in its enhancement.

## 1. Introduction

**Figure 1:**
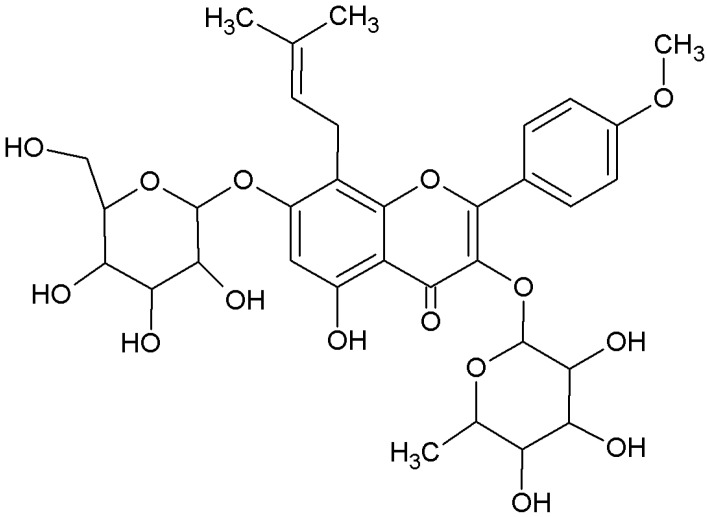
Chemical structure of icariin.

Herba Epimedii, the dried aerial parts of *Epimedium brevicornu* Maxim, *Epimedium sagittatum* Maxim, *Epimedium pubescens* Maxim and *Epimedium koreanum* Nakai, have been traditionally used as tonic, aphrodisiac and antirheumatic medical herbs for cardiovascular, bone loss and impotence diseases in East Asian countries for many years. The major active constituents of Epimedii are flavonoids, of which more than 141 flavonoid compounds, including flavonols, flavones, chalcones, flavanones, and flavonol glycosides, have been isolated [1,2]. Icariin (Figure 1), one of the most abundant flavonoids of Herba Epimedii, has numerous pharmacological and biological activities, including prevention of osteoporosis, erectile impotence, and anti-cancer and anti-depression properties [3,4,5].

In the clinic, *Epimedium* fried with suet oil was usually used, which could enhance the kidney invigorating and “Yang” strengthening roles of *Epimedium* [6]. “Frying” treatment consists of two parts: “heat” and “suet oil”. The role the “heat” factor during processing has been clarified before: heating causes *Epimedium* to produce more absorbable bioactive flavonoids [7]. However, the mechanism of action of how suet oil increases the effects has not been elucidated. 

Sodium deoxycholate (DOC), used in the preparation of self-assembled nanomicelles, is one of the bile acid salts, inherent to the human body. It is a natural biosurfactant, which can promote the preparation of micelles, vesicles and other drug carriers. Bile salts can work with lipids such as phospholipids, glycerides, fatty acids, as well as other surface-active agent to prepare mixed micelles. These mixed micelles can promote a drug’s absorption as the carrier [8].

Suet oil is a kind of fatty oil from *Capra hircus linnaeus* or *Ovis aries linnaeus*, containing unsaturated fatty acids, linoleic acid and saturated fatty acids and so on. Its properties are similar to that of a surfactant, which is helpful for the preparation of nanomicelles. Our experiments found that suet oil can work with DOC to prepare mixed micelles, as the carrier of icariin under simulated gastrointestinal tract conditions. This triggered us to explore how suet oil plays its role in the formulation of nanomicelles, and to explain the relationship between self-assembled nanomicelles with the kidney invigorating and “Yang” strengthening mechanism of *Epimedium* fried with suet oil. We selected icariin as the model flavonoid, and prepared icariin self-assembled nanomicelles with and without suet oil *in vitro*, and characterized them by Malvern Instruments size determination and TEM. The differences between self-assembled nanomicelles with and without suet oil on the transport of icariin across Caco-2 cell monolayers and its absorption in rat intestine were also evaluated and compared. Thus, the action mechanism of suet oil was preliminarily clarified.

## 2. Results and Discussion

### 2.1. Characterization of the Self-Assembled Nanomicelles

#### 2.1.1. Size, Size Distribution and Drug Encapsulation Efficiency

The size, size distribution and drug encapsulation efficiency of the icariin + DOC and icariin + DOC + suet oil prepared in this research are shown in Table 1. The average particle size and polydispersity of icariin + DOC were 553.2 ± 4.06 nm and 0.851 ± 0.010, respectively, while the average particle size of icariin + DOC + suet oil was found to be around 160 nm, with a narrow size distribution of about 0.123 polydispersity. The drug encapsulation efficiency of icariin + DOC + suet oil was 2.08 fold higher than that of icariin + DOC. Therefore, we could conclude that the suet oil used in the fabrication process is an important factor to influence the particle size, size distribution and the drug encapsulation efficiency in the self-assembled nanomicelles.

**Table 1:** Particle size, size distribution and encapsulation efficiency (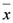 ± S.D., n = 3).

Samples (20 μM)	Average particle size (nm)	Polydispersity	EE (%)
icariin + DOCi	553.2 ± 4.06	0.851 ± 0.010	43.1 ± 1.76
icariin + DOC + suet oili	164.8 ± 0.98 ***	0.123 ± 0.003 ***	89.7 ± 2.23 ***

The number of asterisk symbols indicates the level of significance between the self-assembled nanomicelles of the average particle size, polydispersity and encapsulation efficiency with *** *p* < 0.001.

#### 2.1.2. Surface Morphology

**Figure 2:**
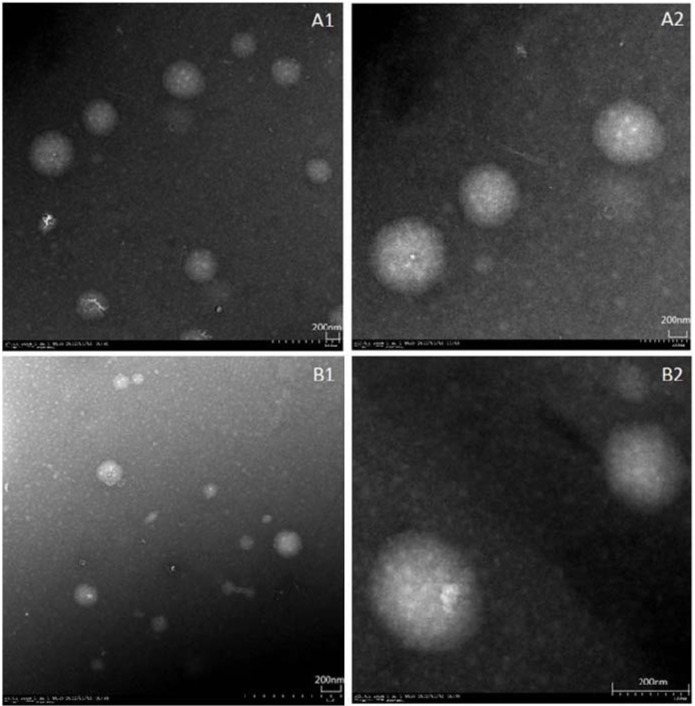
TEM figures of the self-assembled nanomicelles: (**A**) icariin + DOC (A1: ×5.0 K; A2: ×20.0 K); (**B**) icariin + DOC + suet oil (B1: ×7.0 K; B2: ×12.0 K).

The surface morphology of icariin + DOC and icariin + DOC + suet oil were examined by TEM. The self-assembled nanomicelles seemed to be monodisperse spherical particles with smooth surfaces within the TEM resolution level (shown in Figure 2). The TEM images further confirmed the particle size detected by dynamic light scattering (DLS).

### 2.2. Vectorial Transport of icariin + DOC and icariin + DOC + suet oil in Caco-2 Cell Monolayers

**Figure 3:**
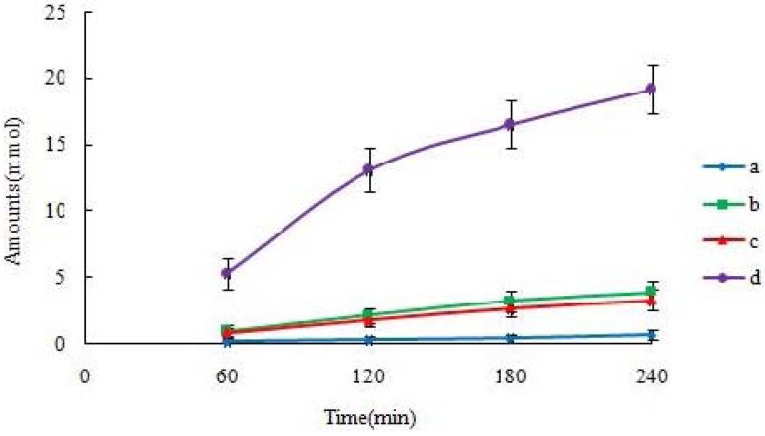
The transportation of icariin as time changes. Cumulative amounts of icariin transported across Caco-2 cell monolayers. The cumulative amount transported at each time point using the Kruskal–Wallis test followed by Tamhane’s post hoc was used to analyze the data statistically, n = 3. (**a**: 20 μM icariin + DOC A-B; **b**: 20 μM icariin + DOC B-A; **c**: 20 μM icariin + DOC + suet oil A-B; **d**: 20 μM icariin + DOC + suet oil B-A).

Icariin + DOC and icariin + DOC + suet oil transported across Caco-2 cell are shown in Figure 3 (each at 20 μM). The absorbed drug (drug in apical loading) increased linearly with time. At the same time, there was also an excretion of drug (drug in basolateral loading). This excretion was apparently larger than absorption. Compared with the icariin + DOC, the icariin + DOC + suet oil increased absorption and promoted excretion of the drug simultaneously on Caco-2 cell monolayers.

**Figure 4:**
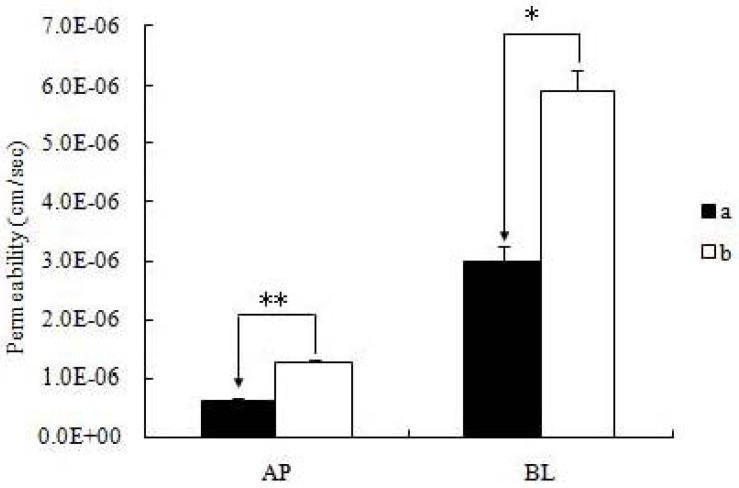
Permeabilities of icariin + DOC and icariin + DOC + suet oil. The experiments were performed at 37°C. Each bar represents the average of three determinations and the error bars are the standard deviation of the means. One-way ANOVA with Tamhane’s *post hoc* test was used to analyze the data statistically, ** *p* < 0.01 and * *p* < 0.05. (**a**: 20 μM icariin + DOC; **b**: 20 μM icariin + DOC + suet oil).

Among the studied self-assembled nanomicelles, icariin + DOC + suet oil was significantly better transported than icariin + DOC (Table 2). As expected, there were significant differences in apical to basolateral (A-B or absorptive) permeabilities between them (*p* < 0.01) (Figure 4). The absorptive permeability of icariin + DOC + suet oil was 2.03 fold higher than icariin + DOC. In contrast, basolateral to apical (B-A or secretory) transport permeabilities of the studied self-assembled nanomicelles were faster than their absorptive transport permeabilities (apical to basolateral transport permeability), respectively (Table 2). The secretory permeability (basolateral to apical transport permeability) of icariin + DOC + suet oil was 5.91 ± 0.38 cm/s, while 3.00 ± 0.27 cm/s was for icariin + DOC. Statistically, there was difference in secretory permeabilities between them (*p* < 0.05) (Figure 3).

**Table 2:** Permeabilities (P) and efflux ratios of the self-assembled nanomicelles (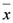 ± S.D., n = 3).

Samples(20 μM)	P_AB_ (cm/s)			P_BA_ (cm/s)			Efflux ratio
	Mean 10^−6^	S.D.10^−7^		Mean 10^−6^	S.D.10^−7^		P_BA_/P_AB_
icariin + DOC	0.62 ± 0.06	0.061		3.00 ± 0.27	0.265		4.84
icariin + DOC + suet oil	1.26 ± 0.03 **	0.035		5.91 ± 0.38 *	0.345		4.69

Experiments were performed in triplicate at 37 °C, pH 7.4 HBSS. Absorptive permeability was expressed as P_AB_, whereas secretory permeability was expressed as P_BA_. One-way ANOVA with Tamhane’s *post hoc* test was used to analyze the data statistically. The number of asterisk symbols indicates the level of significance between the self-assembled nanomicelles of the absorptive permeabilities and secretory permeabilities with ** *p* < 0.01 and * *p* < 0.05.

Efflux ratio of icariin + DOC and icariin + DOC + suet oil were 4.84 and 4.69, respectively. At the end of the transport experiment, integrity of the monolayer was monitored by TEER value, and there was no significant change. These results indicated that the icariin self-assembled nanomicelles with suet oil could enhance the absorption of the drug.

### 2.3. Cellular Accumulation of Icariin + DOC and Icariin + DOC + Suet Oil

**Figure 5:**
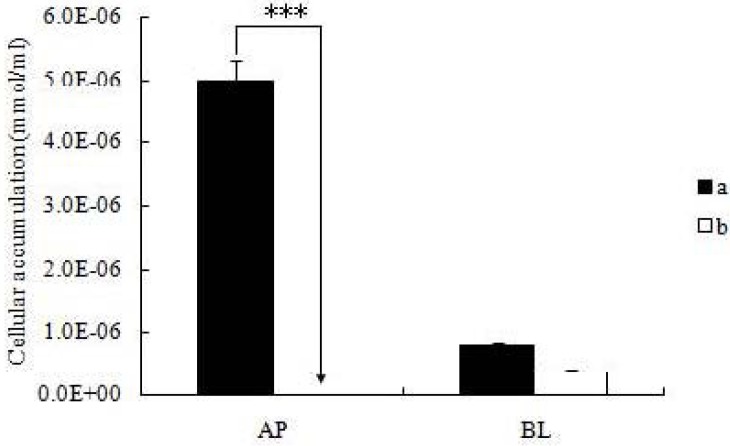
The cellular accumulation of the icariin + DOC and icariin + DOC + suet oil. The accumulation was measured after 4 h incubation of cell monolayers with the icariin + DOC and icariin + DOC + suet oil. Each data point is the average of three determinations, and the error bars represent the standard deviation of the mean. One-way ANOVA with Tamhane’s *post hoc* test was used to analyze the data statistically, *** *p* < 0.001. (**a**: 20 μM icariin + DOC; **b**: 20 μM icariin + DOC + suet oil).

The cellular accumulation of the icariin + DOC and icariin + DOC + suet oil was assessed. The cellular accumulation amounts were (5.00 ± 0.321)E^−6^ mmol/mL and 0 mmol/mL for icariin + DOC and icariin + DOC + suet oil in apical loading (AP loading), (8.02 ± 0.272)E^−7^ mmol/mL and (3.66 ± 0.348)E^−7^ mmol/mL in basolateral loading (BL loading) for icariin + DOC and icariin + DOC + suet oil, respectively. The results showed that there were substantial differences in AP loading between them (*p* < 0.001), while there was no difference in BL loading (Figure 5).

### 2.4. Absorption of Icariin + DOC and Icariin + DOC + Suet oil in Rat Intestine

**Figure 6:**
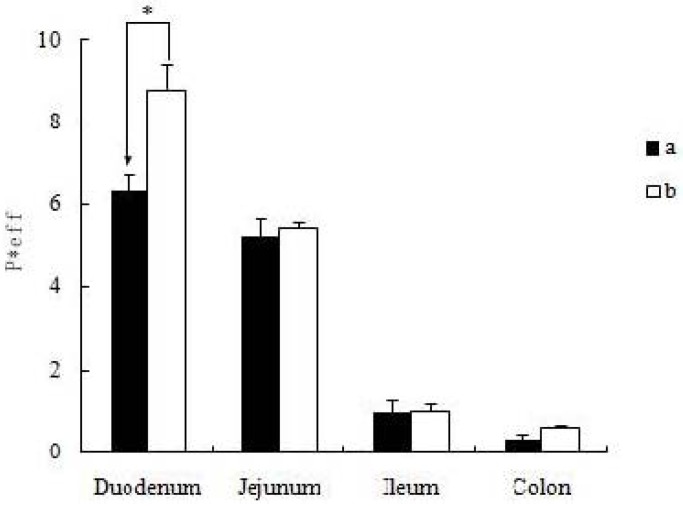
Comparison of permeability coefficient between four different intestinal segments. Data are expressed as mean ± S.D. (n = 4). The statistically significant differences are shown by the asterisk symbol, * *p* < 0.05 (**a**: 20 μM icariin + DOC, **b**: 20 μM icariin + DOC + suet oil).

The P *_eff_ values obtained for the icariin + DOC and icariin + DOC + suet oil following *in situ* perfusion to the different segments are presented in Figure 6. The permeability of icariin + DOC was 6.315 ± 0.437, 5.227 ± 0.444, 0.971 ± 0.279 and 0.325 ± 0.130 in the duodenum, jejunum, ileum and colon, respectively. The permeability of icariin + DOC + suet oil was 8.773 ± 0.609, 5.440 ± 0.122, 0.989 ± 0.179 and 0.595 ± 0.067 in the duodenum, jejunum, ileum and colon, respectively. Based on the data obtained, it appeared that icariin + DOC and icariin + DOC + suet oil exhibited high permeabilities in the duodenum and jejunum. The P *_eff_ value of icariin + DOC + suet oil in duodenum was higher than the value of icariin + DOC (*p* < 0.05) in duodenum. This indicated that the icariin self-assembled nanomicelles formed with the help of suet oil could improve the P *_eff_ value of active icariin, and promote its intestinal absorption.

## 3. Experimental

### 3.1. Materials

Cloned Caco-2 TC7 cells were a kind gift from Dr. Moniqué Rousset of INSERM U178, (Villejuit, France). Icariin (purity > 98%) was purchased from National Institute for the Control of Pharmaceutical and Biological Products (Beijing, China), and sodium deoxycholate was purchased from Sinopharm Chemical Reagent Co., Ltd. (Shanghai, China). Hanks’ balanced salt solution (HBSS; powder form) was purchased from Sigma-Aldrich (St. Louis, MO, USA). Suet oil was provided by Hu Qiao Pharmaceutical Co., Ltd. (Bozhou, China). Milli-Q water (Millipore, Bedford, MA, USA) was used in the study. Acetonitrile was of chromatographic grade (Merck Company Inc., Whitehouse Station, NJ, USA). All other reagents were of analytical grade.

### 3.2. Preparation of the Self-Assembled Nanomicelles

#### 3.2.1. The Preparation of the icariin Self-Assembled Nanomicelles with Suet Oil

To prepare the icariin self-assembled nanomicelles with suet oil, icariin (0.676 mg) was mixed with suet oil (0.1352 mg) and the mixture was put into HBSS buffer (50 mL, pH 7.4) containing DOC (0.676 mg). Then, the solution was sonicated for 10 min at 100% power. The icariin self-assembled nanomicelles with suet oil were abbreviated as icariin + DOC + suet oil. 

#### 3.2.2. The Preparation of the Icariin Self-assembled Nanomicelles without Suet Oil

To prepare the icariin self-assembled nanomicelles without suet oil, icariin (0.676 mg) was dissolved in HBSS buffer (50 mL, pH 7.4) containing DOC (0.676 mg) and the solution was sonicated for 10 min at 100% power. The icariin self-assembled nanomicelles without suet oil were abbreviated as icariin + DOC.

### 3.3. Characterization of the Self-Assembled Nanomicelles

#### 3.3.1. Particle Size, Size Distribution and Surface Morphology

The size and size distribution of self-assembled nanomicelles were determined by dynamic light scattering (DLS) on a Zetasizer Nano-ZS serie MAL1021404 (Malvern Instruments S.A., Worcestershire, UK). The morphological evaluation was performed by transmission electron microscopy (TEM, JEM-1200EX, JEOL Technics Co., Ltd., Akishima, Japan).

#### 3.3.2. The Encapsulation Efficiency of Self-Assembled Nanomicelles

The self-assembled nanomicelles were obtained by ultracentrifugation at 30,000 rpm for 15 min. Then a HPLC assay was used to measure the amount of icariin in the supernatant. The drug entrapment efficiency (E.E.) was expressed as percentage of the icariin difference between the initial amount of icariin and the amount in the supernatant relative to the total amount used for the self-assembled micelle preparation.

E.E. (%) = (1 − amount of icariin in supernatant/amount of icariin added) × 100

The content of icariin in the supernatant was determined by the HPLC method as described below. Approximately 1 mL of the supernatant was dissolved in methanol, and the final volume was 5 mL, then a 20 μL aliquot of the resulting solution was injected into a HPLC system.

### 3.4. Cell Experiments

#### 3.4.1. Cell Culture

The Caco-2 TC7 cell line was a kind gift from the laboratories of Dr. Moniqué Rousset of INSERM U178 (Villejuit, France) and was nominally similar to wild-type Caco-2 cells [9], but it is more stable during passage since it is a cloned cell line. The culture conditions for growing Caco-2 cells have been described previously [10,11,12]. For the transport assay, cells were seeded on top of Transwell inserts, consisting of 3 μm porous polycarbonate, in 6-well Transwell plates, which have a surface area of 4.2 cm^2^. The seeding density (100,000 cells/cm^2^), growth media (Dulbecco’s modified Eagle’s medium supplemented with 10% fetal bovine serum), and quality control criteria were all carried out according to previously published reports [10,11,12]. The Caco-2 TC7 cells were maintained at 37 °C at 90% humidity and 5% CO_2_. The culture media was changed every 48 h. The monolayers, whose ecology and appearance were well developed and the transepithelial electrical resistance (TEER) values were greater than 250 Ω × cm^2^, were ready for experiments between 21 and 23 days [13] after seeding. 

#### 3.4.2. Transport Experiments in the Caco-2 Cell Culture Model

Experiments in triplicate were performed in pH 7.4 HBSS. The protocol for carrying out cell culture experiments was described before [10,11,12]. In short, the cell monolayers were washed three times with 37 °C HBSS. The transepithelial electrical resistance (TEER) values of cell monolayers were measured, which were more than 250 Ω × cm^2^, meeting the requirement of experiment [14]. The monolayers were incubated with the pH 7.4 blank HBSS for 1 h. Then the incubation medium was aspirated. Afterward, a solution containing the icariin was loaded on to the apical or basolateral side. The amount of transported icariin in the receiver media was measured as a function of time using UPLC methods to follow. Donor samples (400 μL) and receiver samples (400 μL) were taken at different times (typically 1 h), followed by the addition of 400 μl of fresh donor solution to the donor side or 400 μL of blank buffer to the receiver side. When comparing the permeability of icariin + DOC and icariin + DOC + suet oil, each sample was used at the same concentration (20 μM) and the samples were taken at 0, 1, 2, 3 and 4 h after incubation. To each transport sample (400 μL), methanol (100 μL) containing 4 μM of genistein was added as an internal standard, and then the resulting mixtures were vortexed for 30 sec, centrifuged at 15,000 rpm for 15 min. The supernatant was analyzed by UPLC method (see below). At the end of the transport experiment, integrity of the monolayer was monitored by TEER value, and there was no significant change [15].

#### 3.4.3. Electrical Resistance

The integrity of the monolayers was determined by measuring the electrical resistance values at the end of each experiment. For this purpose, a Bosstek type instrument with two electrodes was used and the results were expressed as Ω × cm^2^ [16].

#### 3.4.4. Cellular Accumulation Studies in the Cell Monolayers

After the cell monolayers were incubated with icariin + DOC and icariin + DOC + suet oil for a predetermined period of time (4 h), respectively, the mature monolayers were gently washed three times with ice-cold saline immediately after the incubation buffer was removed. Subsequently, the monolayers were cut out together with the porous polycarbonate membranes, put in HBSS buffer (1 mL, pH 7.4), and sonicated in an ice-cold water bath for 30 min using KQ5200DE sonicator (Ultrasonic Instrument Co., Ltd., Kunshan, China) at the maximum power. Then the cellular lysates were centrifuged at 15,000 rpm for 15 min, so the supernatant can be injected into a UPLC instrument.

### 3.5. Animals Experiments

#### 3.5.1. Animals

Male Sprague–Dawley rats weighing between 350 and 400 g were obtained from the SLEK Lab Animal Center of Shanghai (Shanghai, China), housed under standard conditions of temperature, humidity, and light. Food and water were provided *ad libitum*. The rats were fasted overnight before the day of the experiment. 

#### 3.5.2. Animal Surgery

The procedures were approved by the Animal Ethics Committee of Jiangsu Provincial Academy of Chinese Medicine. A day before the experiment, the rats were fasted overnight but were provided deionized water. After overnight fasting, rats were anesthetized. The small intestine was exposed by midline incision; the intestinal lumen was then gently flushed to remove intestinal content and each of the four segments (duodenum, upper jejunum, terminal ileum, and colon) of the intestine was cannulated with two cannulaes. The outlet of each segment was secured by ligation with silk suture. The intestine was carefully arranged and continuously monitored to avoid kinks and ensure a consistent flow after cannulation. Saline-soaked cotton was used to cover opened body cavities to prevent loss of fluids [17].

#### 3.5.3. Four Site Single-pass Rat Intestinal Perfusion Experiments

A single-pass perfusion method was used by perfusing the small intestine as one whole segment (from duodenum to ileum). To keep the temperature of the perfusate constant, the inlet cannulae was insulated and kept warm by a 37 °C circulating water bath. A flow rate of 0.412 mL/min was used, and the perfusate samples were collected every 30 min. The outlet concentration of drug in the perfusate was determined by UPLC.

### 3.6. Analytical Methods

UPLC method: The UPLC method was used to detect the icariin in the transport samples obtained from the Caco-2 model and the single-pass rat intestinal perfusion experiment. The conditions for UPLC analysis of icariin were as follows: Waters Acquity UPLC system with photodiode array detector and Empower software; column, Acquity UPLC BEH C18, 1.7 μm, 2.1 × 50 mm (Waters, Milford, MA, USA); mobile phase A, acetonitrile; mobile phase B, water containing 0.1% HCOOH; gradient, 0 to 0.5 min, 10% A, 0.5 to 1 min, 10% to 40% A, 1 to 2.5 min, 40% A, 2.5 to 3 min, 40% A to 100% A, 3 to 4 min, 10% A; flow rate, 0.4ml/min; colume temperature, 35 °C; wavelength, 270 nm; injection volume, 8 μL. The retention time for icariin is 1.395 min. The retention time for genistein as an internal standard is 1.590 min. In general, the method is selective and reproducible with day to day variability less than 2.5%. The accuracy and precision were greater than 98%. The tested linear response ranges for samples were 0.3125 to 40 μM.

HPLC method [6]: The stationary phase, a Phenomenex® C18 (4.6 × 150mm, 5 μm) column, was used and kept at 30 °C. The mobile phase was a mixture of acetonitrile-water (75:25). The flow rate was 1.0 mL/min. Separation was monitored at 270 nm. The tested linear response ranges for samples were 0.02 to 0.64 μM.

### 3.7. Data Analysis

Rate of the transportation of icariin is obtained from amount transported *versus* time curve using linear regression. The apparent permeability (Papp) of icariin is calculated using the following equation:

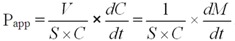
(1)
In the equation, *V* is the volume of the receiver (typical volume is 2.5 mL), *S* is the surface area of the cell monolayer (typical surface area is 4.2 cm^2^), *C* is the initial concentration, *dC/dt* is the rate of concentration change in the receiver side, and *dM/dt* is the rate of drug transport. The rate of drug transport is obtained by linear regression analysis (a Microsoft Excel function). 

The absorption of drug was measured by the rate of its disappearance, and the permeability coefficient (P*eff) was determined through the rate of disappearance. The effective P*eff was calculated using the following equation:

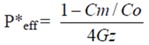
(2)
In the equation, *Cm* is the concentration of the permeant in the exiting perfusate; *Co* is the concentration of the permeant in entering perfusate; *Gz*, or Graetz number (*Gz = πDL/*2*Q*), is a scaling factor that incorporates flow rate (Q) and intestinal length (L), and diffusion coefficients (D) to make the permeability dimensionless. The experimental data was put into the formula, and the result could be obtained by the designed excel table. Values were indicated as mean ± S.D. for permeability in four independent rats.

### 3.8. Statistical Analysis

All experiments were conducted at least in triplicate. Data was presented as mean ± S.D. Student’s t test (Microsoft Excel) was used to analyze the data when there were only two groups in the experiments. A two-tailed t-test (*p* < 0.01) was used to identify significant differences of permeability results between the icariin + DOC group and icariin + DOC + suet oil group.

## 4. Conclusions

The DOC can promote the formation of micelles. Therefore, the icariin self-assembled nanomicelles without suet oil can be formed. However, the suet oil has a fatty acid composition, which have long fatty chains and surfactant properties. The self-assembled nanomicelles prepared with suet oil have smaller size than those without suet oil, and are more stable. Under the joint action of suet oil and DOC, self-assembled hybrid nanomicelles could be prepared. The formulated carrier could promote the drug’s absorption [18,19]. The self-assembled nanomicelles with suet oil have smaller polydispersity and higher drug encapsulation efficiency than that without suet oil. In Caco-2 cell monolayers, the absorptive permeability of icariin + DOC + suet oil was 2.03 fold higher than that of icariin + DOC. In the four site single-pass rat intestinal perfusion experiment, there was statistical difference in the permeability value between icariin + DOC and icariin + DOC + suet oil in duodenum. 

When the *Epimedium* fried with suet oil is taken orally into the body, suet oil could work together with DOC, inherent in the human body, to form mixed micelles. Icariin is one of the most important flavonoids of *Epimedium*, whose absorption can be promoted through the carrier. Thus, a tentative interpretation of the enhancement of activity seen with *Epimedium* fried with suet oil can be provided.

## References

[1] Chen Y., Wang J.Y., Jia X.B., Tan X.B., Hu M. (2011). Role of Intestinal Hydrolase in the Absorption of Prenylated Flavonoids Present in Yinyanghuo. Molecules.

[2] Wei Y.J., Li P., Fan H.W., Sun E., Wang C.M., Shu L., Liu W., Xue X.L., Qian Q., Jia X.B. (2012). Metabolite Profiling of Four Major Flavonoids of Herba Epimdii in Zebrafish. Molecules.

[3] Fan J.J., Cao L.G., Wu T., Wang D.X., Jin D., Jiang S., Zhang Z.Y., Bi L., Pei G.X. (2011). The Dose-Effect of Icariin on the Proliferation and Osteogenic Differentiation of Human Bone Mesenchymal Stem Cells. Molecules.

[4] Huang X., Zhu D.Y., Lou Y.J. (2007). A novel anticancer agent, Icaritin, Induced cell growth inhibition, G1 arrest and mitochondrial transmembrane potential drop in human prostate carcinoma PC-3 cells. Eur. J. Pharmacol..

[5] Ning H., Xin Z.C., Lin GT., Banie L., Lue T.F., Lin C.S. (2006). Effects of icariin on phosphodiesterase-5 activity *in vitro* and cyclic guanosine monophosphate level in cavernous smooth muscle cells. Urology.

[6] Editorial Committee of Pharmacopoeia of Ministry of Health PR China (2010). The Pharmacopeoia of People’s Republic of China.

[7] Chen Y., Zhao Y.H., Jia X.B., Hu M. (2008). Intestinal Absorption Mechanisms of Prenylated Flavonoids Present in the Heat-Processed *Epimedium koreanum* Nakai (Yin Yanghuo). Pharm. Res..

[8] Jin Y.G., Tong L., Ai P., Li M., Hou X.P. (2006). Self-assembled drug delivery systems: 1. Properties and *in vitro/in vivo* behavior of acyelovir self-assembled nanoparticles(SAN). Int. J. Pharm..

[9] Lin J.B., Dou J., Xu J.L., Haji A.A. (2012). Chemical Composition, Antimicrobial and Antitumor Activities of the Essential Oils and Crude Extracts of *Euphorbia* macrorrhiza. Molecules.

[10] Chen J., Lin H., Hu M. (2003). Metabolism of flavonoids via enteric recycling: Role of intestinal disposition. J. Pharmacol. Exp. Ther..

[11] Hu M., Chen J., Lin H. (2003). Metabolism of flavonoids via enteric recycling: mechanistic studies of disposition of apigenin in the Caco-2 cell culture model. J. Pharmacol. Exp. Ther..

[12] Hu M., Chen J., Zhu Y., Dantzig A.H., Stratford R.E., Kuhfeld M.T. (1994). Mechanism and kinetics of transcellular transport of a new beta-lactam antibiotic loracarbef across an intestinal epithelial membrane model system (Caco-2). Pharm. Res..

[13] Karamustafa F. Transport of alendronate through human intestinal cell line, Caco-2. Proceedings of the 33rd Annual Meeting & Exposition of the Controlled Release Society.

[14] Roger E., Lagarce F., Garcion E., Benoit J.P. (2009). Lipid nanocarriers improve paclitaxel transport throughout human intestinal epithelial cells by using vesicle-mediated transcytosis. J. Control. Release.

[15] Lev B., Valery A. (2003). Effects of polyether-modified poly (acrylic acid) Microgels on doxorubicin transport in human intestinal epithelial Caco-2 cell. J. Control. Release.

[16] Lo Y.L. (2003). Relationships between the hydrophilic–lipophilic balance values of pharmaceutical excipients and their multidrug resistance modulating effect in Caco-2 cells and rat intestines. J. Control. Release.

[17] Abuasal B., Sylvester P.W., Kaddoumi A. (2010). Intestinal Absorption of -Tocotrienol Is Mediated by Niemann-Pick C1-Like 1: *In Situ* Rat Intestinal Perfusion Studies. Drug Metab. Dispos..

[18] van Hasselt P.M., Janssens G.E.P.J., Rijcken C.J.F., van Nostrum C.F. (2008). Influence of bile on the oral bioavailability of vitamin K-loaded polymeric micelles. J. Control. Release.

[19] Jiang L.X., Wang K., Deng M.L., Wang Y.L., Huang J.B. (2008). Bile salt-induced vesicle-to-micelle transition in catanionic surfactant systems: Steric and electrostatic interactions. Langmuir.

